# The predictive accuracy of axial length to corneal curvature radius ratio for myopia in children and adolescents

**DOI:** 10.3389/fmed.2026.1758072

**Published:** 2026-04-21

**Authors:** Lin Wang, Qianru Zhang, Changdong Liu, Qiqi Liu, Peipei Zhang

**Affiliations:** 1 Department of Ophthalmology, Affiliated Hospital of Binzhou Medical University, Binzhou, Shandong, China; 2 Department of Ophthalmology, Yantai Aier Ophthalmology Hospital, Yantai, Shandong, China; 3 Department of Ophthalmology, Shenzhen Guangming District People’s Hospital, Shenzhen, Guangdong, China

**Keywords:** axial length, axial length to corneal curvature radius ratio, children and adolescents, corneal curvature radius ratio, myopia

## Abstract

**Purpose:**

To analyze the association of axial length to corneal curvature radius ratio (AL/CR) and other ocular biometric parameters with myopia in children and adolescents.

**Methods:**

A cross-sectional study was conducted among children and adolescents aged 6 to 12 years without ocular diseases who attended the optometry clinic of the Yantai Aier Ophthalmology Hospital from February 2022 to February 2024. The visual acuity, ocular biometry, and automatic refraction under cycloplegia were measured. The axial length, corneal curvature radius, and AL/CR were compared across different age groups. Pearson correlation and linear regression were performed to analyze the effects of correlated factors on spherical equivalent. Receiver operating characteristic curves were constructed to evaluate the diagnostic efficacy in detecting myopia.

**Results:**

A total of 2,760 eyes (right eye) of 2,760 children and adolescents were included. Both axial length and AL/CR increased with age in children and adolescents aged 6 to 12 years, while spherical equivalent decreased with age. Corneal curvature radius did not exhibit significant changes with age. The correlation coefficients between AL/CR, axial length, and spherical equivalent are −0.865 and −0.747, respectively, whereas axial length shows no significant correlation (*r* = −0.083). Upon stratification by age, the correlation between AL/CR and spherical equivalent remains stronger than that between axial length and spherical equivalent. The linear regression models for predicting spherical equivalent based on AL/CR, axial length, and age are as follows: spherical equivalent = 36.463–12.278 * AL/CR and spherical equivalent = 28.835–1.260 * axial length. The area under the receiver operating characteristic curve for the AL/CR is 0.938, which surpasses that of axial length at 0.87. The AL/CR cutoff point was 3.026, and the sensitivity and specificity were 0.898 and 0.826, respectively.

**Conclusion:**

The correlation between AL/CR and spherical equivalent (SE) is stronger than that between AL and SE in children and adolescents. AL/CR can independently reflect dynamic changes in SE during myopia progression, without relying solely on optometric data.

## Introduction

The prevalence of myopia, particularly high myopia, has emerged a significant public health concern, increasingly affecting young children (1, 2). Over the past decade, the prevalence of myopia has risen by 23% among East Asians (3). A systematic review and meta-analysis have demonstrated a substantial increase in myopia rates in China over recent decades, with projections indicating a continued rise in prevalence from 2020 to 2050. The study reveals that the overall prevalence of myopia in children is 36.6%, with high myopia affecting 5.3% of the population. Projections suggest that by 2050, the prevalence of myopia and high myopia could reach 61.3 and 17.6%, respectively, under certain scenarios, highlighting the urgent need for effective prevention and control measures (4).

The age of myopia onset may be the most reliable predictor of high myopia (5). Research indicates that the risk of high myopia is relatively high in children who develop myopia during early school years. Each year of delay in the age of onset significantly reduces the chance of developing high myopia in adulthood, highlighting the importance of identifying effective prevention strategies under investigation (6). Over the past 16 years, the average age of myopia onset in Chinese children has decreased by three years (7). Additionally, school closures during the COVID-19 pandemic have heightened the risk of myopia among Chinese children and adolescents, attributed to the accumulation of poor eyesight habits, unhealthy lifestyles, and excessive screen time (8).

The axial length to corneal curvature radius ratio (AL/CR) has emerged as a critical biometric parameter for understanding myopia, particularly in its correlation with refractive errors and its potential predictive value for the onset and progression of myopia in young individuals. A study focusing on adult myopia patients revealed that both axial length (AL) and AL/CR were significantly negatively correlated with spherical equivalent (SE), with the correlation between AL/CR and spherical equivalent (SE) being stronger than that between AL and SE. Moreover, the diagnostic value of AL/CR for high myopia was found to be superior to that of AL (*p* < 0.01) (9). In pediatric populations, AL/CR has also demonstrated promise as a predictive tool. Research conducted among Chinese children indicated that the correlation between SE and AL/CR was stronger than that between SE and AL (10). The study suggests that the AL/CR ratio could be a useful indicator for assessing myopia and emphasizes its potential in early detection and intervention for children.

However, large-scale research on its predictive accuracy is scarce, and its effectiveness in tracking myopia progression in young people needs more validation. This study will gather clinical data from 6–12-year-olds, analyze refractive parameters, and assess the AL/CR’s role in predicting myopia. It will also establish age-specific cutoff values for diagnosing refractive errors.

## Materials and methods

This single-center, cross-sectional study was conducted according to the tenets of the Declaration of Helsinki. Children and adolescents aged 6–12 years who visited the outpatient department of Yantai Aier Ophthalmology Hospital between February 2023 and February 2024 were enrolled. Exclusion criteria were (1) patients with corneal diseases, fundus diseases, glaucoma, refractive media opacities, or other ocular pathologies; (2) patients unable to cooperate with the examination procedures; (3) patients experiencing adverse reactions following pupil dilation.

### Ocular examination and data collection

A standardized ocular examination was performed on each participant. The protocol began with the recording of demographic information (name, age, and gender), followed by a series of assessments including uncorrected distance visual acuity (UCVA), slit-lamp examination, cycloplegic refraction using an automatic refractometer (RM-800; Topcon), and ocular biometry with an optical biometer (IOL Master 500).

Cycloplegia was induced by administering 1% cyclopentolate hydrochloride eye drops at 5-min intervals for a total of 3–5 drops. Refractive error was measured using the automatic refractometer 40 min after the initial instillation. Spherical power (DS) and cylindrical power (DC) were recorded, and SE was calculated as SE = DS + 0.5 × DC. Myopia was defined as SE ≤ −0.50 diopters (D), emmetropia as −0.50 D < SE < +0.50 D, and hyperopia as SE ≥ +0.50 D.

Ocular biometric parameters, including axial length (AL) and horizontal and vertical corneal curvature (K1 and K2), were obtained using the optical biometer. Mean corneal curvature (K) was derived as (K1 + K2)/2, and the corneal radius of curvature (CR) was calculated as CR = 1,000 × (1.3375 − 1)/K, where 1.3375 represents the corneal refractive index.

### Statistical analysis

Statistical analyses were performed using SPSS 19.0 (IBM, Chicago, IL, United States). The normality of the data distribution was examined using the Shapiro–Wilk test. Continuous variables that followed a normal distribution are expressed as mean ± standard deviation (SD). Participants were stratified into seven age groups (6–12 years). Intergroup differences in AL, CR, AL/CR, and SE were assessed using one-way analysis of variance (ANOVA), followed by the Games–Howell *post-hoc* test for pairwise comparisons. Pearson correlation and linear regression were performed to analyze the effects of correlated factors on SE. Receiver operating characteristic (ROC) curves were constructed to evaluate the diagnostic performance of AL and AL/CR in detecting myopia, using cycloplegic SE as the reference standard. The area under the curve (AUC) was calculated as a measure of predictive accuracy. A *p*-value <0.05 was considered statistically significant.

## Results

A total of 2,760 eyes were collected. Among them, there were 1,403 males (50.85%) and 1,357 females (49.15%). Age distribution from 6 to 12 years was 221 (8%), 368 (13.3%), 290 (10.5%), 608 (22.0%), 440 (15.9%), 485 (17.6%), and 348 (12.6%) cases, respectively. The demographic and ocular characteristics of the participants are summarized in Table 1. The AL demonstrated a progressive increase with age, rising from 22.55 ± 1.09 mm at 6 years to 24.62 ± 1.04 mm by 12 years. The average annual growth rate was 0.5 mm between ages 6–9 and 0.3 mm between ages 9–12. Similarly, the AL/CR increased from 2.91 ± 0.13 at 6 years to 3.15 ± 0.13 at 12 years. The SE decreased with age, shifting from 0.29 ± 2.25 D at 6 years to −2.24 ± 1.97 D by 12 years. Notably, the mean SE was −0.54 ± 1.95 D at 8 years, indicating a shift from hyperopia toward myopia. No significant age-related changes were observed in CR.

**Table 1:** The demographic and ocular characteristics of the participants.

Age (years)	6	7	8	9	10	11	12	*F* ^a^	*p*
AL (mm)	22.55 ± 1.09	23.06 ± 0.99^*^	23.48 ± 1.07^*^	23.87 ± 0.94^*^	24.15 ± 1.05^*^	24.37 ± 1.05^*^	24.62 ± 1.04^*^	162.735	<0.001
CR (mm)	7.74 ± 0.24	7.74 ± 0.25	7.78 ± 0.24	7.77 ± 0.25	7.77 ± 0.25	7.80 ± 0.27	7.82 ± 0.25^*^	3.892	0.001
AL/CR	2.91 ± 0.13	2.98 ± 0.12^*^	3.02 ± 0.13^*^	3.08 ± 0.12^*^	3.11 ± 0.12^*^	3.13 ± 0.13^*^	3.15 ± 0.13^*^	152.964	<0.001
SE (D)	0.29 ± 2.25	−0.22 ± 1.67	−0.54 ± 1.95^*^	−1.25 ± 1.72^*^	−1.56 ± 1.85^*^	−1.86 ± 1.82^*^	−2.24 ± 1.97^*^	78.301	<0.001

AL, axial length (mm); CR, corneal radius of curvature (mm); AL/CR, axial length to corneal curvature radius ratio; SE, spherical equivalent (D).

^*^p < 0.05 compared with the age-6 group using the Games–Howell *post-hoc* test.

a One-way ANOVA.

**Figure 1:**
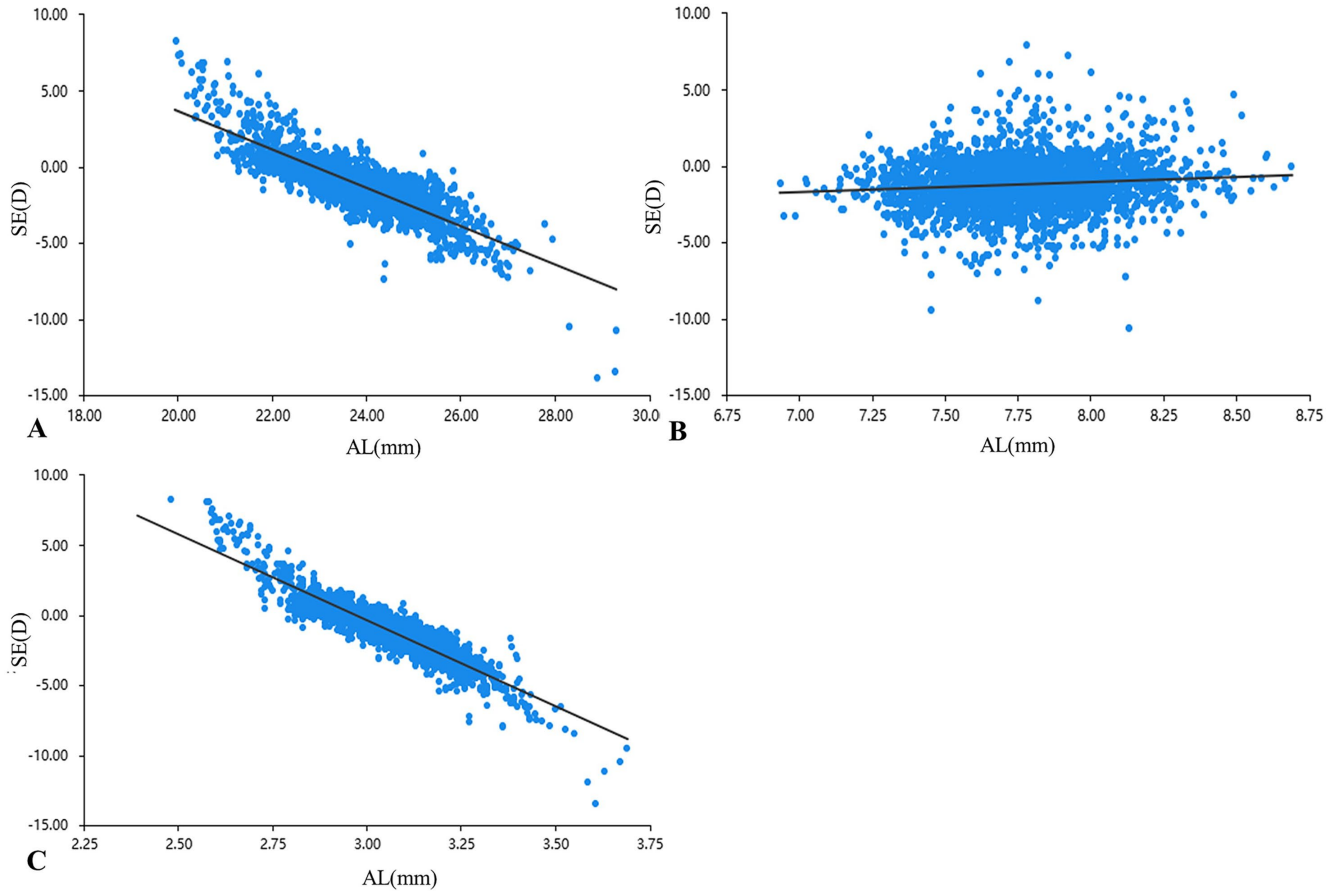
Relation between AL **(A)**, CR **(B)**, AL/CR **(C)**, and SE. AL, axial length (mm); CR, corneal radius of curvature (mm); AL/CR, axial length to corneal curvature radius ratio, SE, spherical equivalent (D).

**Figure 2:**
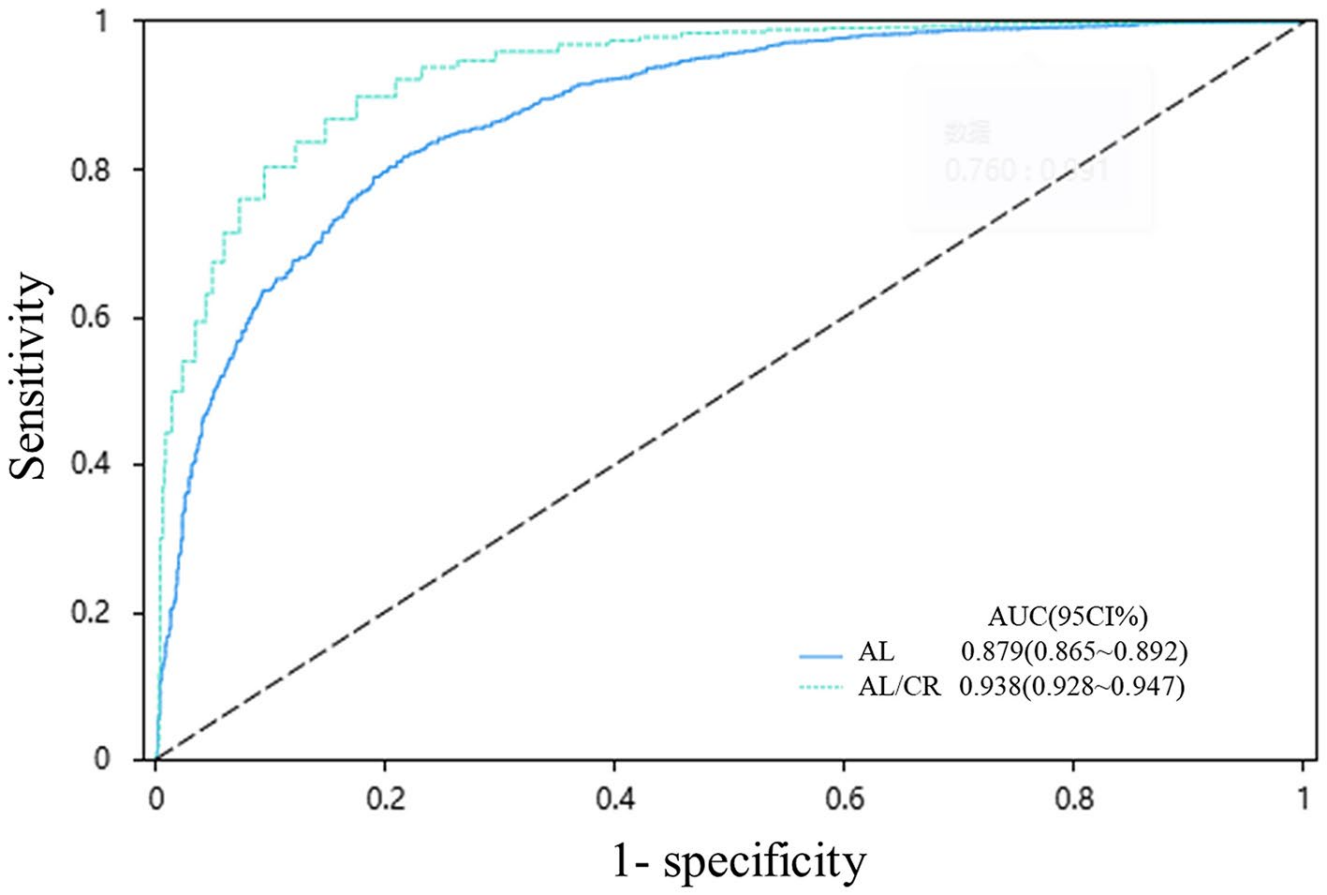
ROC curve of AL and AL/CR in the diagnosis of myopia. AL, axial length (mm); AL/CR, axial length to corneal curvature radius ratio.

The absolute values of the correlation coefficient (*r*) for AL across all age groups ranged from 0.6 to 0.8, indicating a strong correlation with SE. In contrast, the AL/CR showed even stronger associations: with the exception of the 7-year-old group (*r* = −0.789, strong correlation), all other age groups exhibited *r* values between 0.8 and 1.0, reflecting very strong correlations (Table 2). Figure 1 presents the relationship between SE and the other biometric parameters. Liner regression analysis revealed the following equations: SE = 36.463–12.278 * AL/CR, SE = 28.835–1.260 * AL, SE = −6.353 + 0.663 * CR, respectively. The AUC of AL/CR for predicting myopia was 0.938 (95% CI: 0.928–0.947), significantly higher than that of AL (AUC = 0.879, 95% CI: 0.865–0.892) (Figure 2). When the Youden index reached its maximum (0.724), the AL/CR cutoff point was 3.026, and the sensitivity and specificity were 0.898 and 0.826, respectively. Age-stratified analysis revealed a progressive increase in the optimal AL/CR diagnostic cutoff point with advancing age (Table 3).

**Table 2:** Correlation analysis of AL and AL/CR with SE: overall and age-stratified comparisons.

Age (years)	*N*	AL		AL/CR	
		*r* ^a^	*p*	*r* ^a^	*p*
6	221	−0.743	<0.001	−0.858	<0.001
7	368	−0.661	<0.001	−0.789	<0.001
8	289	−0.725	<0.001	−0.867	<0.001
9	607	−0.655	<0.001	−0.836	<0.001
10	440	−0.695	<0.001	−0.850	<0.001
11	484	−0.703	<0.001	−0.841	<0.001
12	347	−0.700	<0.001	−0.870	<0.001
Total	2,760	−0.747	<0.001	−0.865	<0.001

AL, axial length (mm); AL/CR, axial length to corneal curvature radius ratio; SE, spherical equivalent (D).

a Pearson correlation.

**Table 3:** Age-stratified AL/CR diagnostic cutoff point for myopia.

Age (years)	6	7	8	9	10	11	12	Total
Youden’s index	0.727	0.709	0.697	0.732	0.647	0.707	0.620	0.724
Specificity	0.966	0.848	0.881	0.908	0.825	0.848	0.829	0.826
Sensitivity	0.761	0.861	0.816	0.815	0.822	0.859	0.792	0.898
Cutoff point	2.980	2.999	3.025	3.051	3.058	3.068	3.086	3.026
AUC	0.919	0.908	0.909	0.927	0.905	0.919	0.905	0.938
95% confidence intervals	0.867–0.971	0.877–0.94	0.874–0.944	0.903–0.95	0.87–0.941	0.891–0.947	0.862–0.948	0.928–0.947
*p*-value	<0.001	<0.001	<0.001	<0.001	<0.001	<0.001	<0.001	<0.001

AL/CR, axial length to corneal curvature radius ratio; AUC, the area under the curve.

ROC curve analysis was used as the statistical method.

## Discussion

This study concentrated on children aged 6–12 years, a pivotal period for visual development, and examined ocular biometric parameters across different age groups. We assessed the predictive accuracy of AL, CR, and the AL/CR for myopia, establishing age-specific AL/CR cutoff values for diagnosing refractive errors, thereby providing a reference for individualized clinical evaluation.

Previous studies have identified significant variations in AL, AL/CR, and CR among groups with varying degrees of myopia (11, 12). This indicates that age may substantially influence the distribution of ocular biological parameters. Our findings revealed significant differences in SE, AL/CR, AL, and CR across various age groups, suggesting that older children within the 6–12 age range exhibit smaller SE values but larger AL/CR, AL, and CR measurements. These results are consistent with prior studies (13, 14). AL and CR in children undergo continuous changes as ocular development advances. Our results also indicated a transition from hyperopia to emmetropia, with the onset of myopia occurring around the age of 8. This transition is a well-documented phenomenon in ocular development, characterized by alterations in refractive error and AL, which are crucial for understanding the progression of myopia in children. Notably, the age at which myopia onset occurs may serve as the most reliable predictor of high myopia (5).

The corneal refractive power is predominantly determined by the CR, with a smaller CR being associated with increased corneal refractive power. AL and CR are two essential biological determinants of the eye’s refractive status (15). The relationship between the AL/CR and ametropia was initially proposed by Grosvenor (16). In this study, the Pearson correlation coefficient was used to analyze the relationships between AL, AL/CR, and SE. The results demonstrated that while both AL and AL/CR were negatively correlated with SE, the correlation between AL/CR and SE was more pronounced than that between AL and SE across various age groups. This suggests that the combination of AL and CR serves as a more reliable predictor of SE than AL alone. These results are consistent with those reported in previous studies (2, 9, 13). Further analysis of the correlations between AL, CR, AL/CR, and SE was conducted using linear regression. Specifically, for each unit increase in AL/CR, SE decreased by 12.278 D, indicating a strong negative correlation between these variables. AL/CR may serve as a more stable indicator for assessing the progression of myopia.

To further evaluate the diagnostic efficacy of AL/CR in myopia, a ROC curve analysis was employed. This analysis confirmed the superior diagnostic accuracy of AL/CR compared to AL alone, as evidenced by a larger AUC of 0.938 versus 0.879. The findings indicated that the sensitivity and specificity of AL/CR for predicting myopia were 0.898 and 0.826, respectively. This superiority of AL/CR is consistent with recent large-scale and ethnically specific studies. For instance, a large-scale study by Chen et al. (17) reported a significantly larger AUC for myopia detection using AL/CR (0.9112) compared to AL alone (0.8923, *p* < 0.001). Similarly, a hospital-based study involving South-Indian children demonstrated that AL/CR could account for 71% of the total variance in SE and exhibited a stronger correlation with SE (rho = −0.83) than AL alone (rho = −0.68) (18). In our study, the cutoff values for predicting myopia using the AL/CR increased with age, ranging from 2.980 in 6-year-olds to 3.086 in 12-year-olds. These findings are in close alignment with previous studies (17, 19). Chen et al. (17) also reported age-related increases in AL/CR cutoffs, ranging from >2.755 at age 3 to >3.095 at age 18, with gender-specific variations indicating higher cutoffs for boys compared to girls at equivalent ages. Notably, at age 8—a critical transition point where the mean SE reached −0.54 ± 1.95 D—the mean AL/CR was 3.025 ± 0.13, closely aligning with the group-specific cutoff of 3.025. This observation underscores the utility of an AL/CR value of 3.026 as a practical marker for identifying the onset of myopia. An AL/CR exceeding 3.026 may serve as an early warning indicator for myopia, facilitating timely intervention to decelerate its progression and ultimately mitigate the risk of ocular diseases associated with high myopia.

The AL/CR offers a straightforward, rapid, and precise method for predicting myopia in children and adolescents. As an objective measure, it is less influenced by subjective or accommodative factors, and is more amenable to cooperation from children during examinations. Importantly, this method holds clinical significance in predicting the onset and progression of myopia in children who are either unwilling or unable to undergo dilated optometry (13). Vohnsen (20) suggested that emmetropization relies on a delicate balance between photoreceptor outer segment length and density in relation to pupil size. When abnormal eye growth occurs, reflected by an increased AL/CR ratio, crosstalk between adjacent outer segments may not be effectively suppressed, thereby reducing the highest possible light capture efficiency of visual pigments in the outer segments, which in turn drives myopia. A basic differential model (21) offers a theoretical framework for why the AL/CR ratio serves as such a powerful clinical tool: emmetropization and ocular homeostasis rely on a delicate balance between axial length and optical power—the latter comprising the combined powers of the cornea and lens, for which corneal curvature radius is a major determinant. Previous research has established that peripheral defocus influences the regulation of axial length growth (22). Specifically, hyperopic defocus is associated with axial elongation, whereas myopic defocus tends to inhibit this growth (23). For individuals with a larger AL/CR, which indicates a greater curvature of the posterior eyeball relative to the cornea, incoming light is more likely to induce hyperopic defocus around the retina (9). This relationship may contribute to the AL/CR ratio’s effectiveness as a diagnostic tool for identifying individuals at risk of developing myopia.

In addition to its diagnostic value, the AL/CR may also be relevant for monitoring the efficacy of myopia control interventions. A retrospective study conducted in 2025 compared the effects of 0.01% low-dose atropine (LA) and orthokeratology (OK) on AL elongation, finding that both treatments significantly reduced AL elongation compared to single-vision spectacles (SV) over a one-year period (24). Given that AL is a critical component of the AL/CR, the effectiveness of these interventions in controlling axial elongation should logically be reflected in alterations to the AL/CR. Future research that directly incorporates the AL/CR could potentially employ this parameter as a more comprehensive biomarker for assessing and comparing treatment outcomes.

This study has several limitations. Its cross-sectional design restricts the ability to draw longitudinal inferences about refractive changes prior to the onset of myopia. Consequently, the establishment of a causal relationship between AL/CR and myopia remains constrained. Selection bias related to geographic, ethnic, or socioeconomic factors could limit generalizability. Furthermore, although all participants were recruited from the Yantai area, where children generally share similar lifestyles due to consistent regional educational policies and cultural practices, we did not collect detailed data on lifestyle factors such as time outdoors, dietary habits, or daily routines. These factors are known to influence myopia development. Future studies should incorporate these variables to improve predictive accuracy.

In summary, this study demonstrated a significant negative correlation between both AL and AL/CR with SE, with AL/CR exhibiting a stronger correlation with SE than AL alone. These findings reinforce AL/CR as an independent factor associated with myopia development in children and adolescents. Further large-scale, multicenter studies are warranted to validate these results and facilitate the clinical translation of AL/CR as a reliable, non-invasive, and widely applicable alternative method for refractive assessment.

## Data Availability

The original contributions presented in the study are included in the article/supplementary material, further inquiries can be directed to the corresponding author.
